# The effects of food advertising and cognitive load on food choices

**DOI:** 10.1186/1471-2458-14-342

**Published:** 2014-04-10

**Authors:** Frederick J Zimmerman, Sandhya V Shimoga

**Affiliations:** 1 Department of Health Policy & Management, Fielding School of Public Health, UCLA, Los Angeles, CA, USA

**Keywords:** Behavioral economics, Advertising, Obesity, Food choice, Cognitive load

## Abstract

**Background:**

Advertising has been implicated in the declining quality of the American diet, but much of the research has been conducted with children rather than adults. This study tested the effects of televised food advertising on adult food choice.

**Methods:**

Participants (N = 351) were randomized into one of 4 experimental conditions: exposure to food advertising vs. exposure to non-food advertising, and within each of these groups, exposure to a task that was either cognitively demanding or not cognitively demanding. The number of unhealthy snacks chosen was subsequently measured, along with total calories of the snacks chosen.

**Results:**

Those exposed to food advertising chose 28% more unhealthy snacks than those exposed to non-food-advertising (95% CI: 7% - 53%), with a total caloric value that was 65 kcal higher (95% CI: 10-121). The effect of advertising was not significant among those assigned to the low-cognitive-load group, but was large and significant among those assigned to the high-cognitive-load group: 43% more unhealthy snacks (95% CI: 11% - 85%) and 94 more total calories (95% CI: 19-169).

**Conclusions:**

Televised food advertising has strong effects on individual food choice, and these effects are magnified when individuals are cognitively occupied by other tasks.

## Background

The quality of the typical American diet has been eroding for decades, a development that some researchers have associated with the growth in food marketing [1-3]. Although each of the “4 P’s” of marketing—product [4], place [5], price [6,7], and promotion [8]—have contributed to the erosion of the American diet, that part of promotion that comprises television advertising has certainly played a significant role [9-14]. Even among adults, food advertising has strong effects [9,15-18].

Recent research in psychology and behavioral economics has shown that cognitive resources are inherently limited [19,20]. People are able to make attentive, rational-seeming decisions some of the time, but at other times decisions seem to be irrational, relying on such cognitive shortcuts as heuristics, social referencing, and habit [21-24].

In the particular area of behaviors around what and how much we eat, people seem to be so sensitive to such effects that eating itself has been described as an “automatic behavior” [25]. In several recent experiments, researchers have discovered that portion size, the behavior of nearby eaters, the accessibility of food, and even dubious health claims all affect the amount and types of food consumed [26-29].

A separate strand of research has shown that eating behaviors are sensitive to the depletion of cognitive resources at any given time (that is, to cognitive load). One study manipulated available cognitive resources by asking participants to memorize either a 2-digit or a 7-digit number, walk down a hallway to another room, and recall the number [30]. Along the way, participants were offered the choice of a chocolate cake or a fruit salad. Among those who had been given a 7-digit number 63% chose the chocolate cake, whereas among those remembering a 2-digit number only 41% chose the cake. Another study produced similar results among restrained eaters [31]. These results were accentuated when the cognitive load represented an ego threat to the participant [32].

There accordingly appears to be strong evidence that eating behaviors are highly sensitive to external cues (including advertising), and cognitive load tends to disinhibit eating. Putting these two strands of research together suggests that the effects of food advertising may be greater among those under a heavy cognitive load than among those whose cognitive resources are not so taxed.

Recent work has shown that foods of low nutritional quality are more heavily marketed in low-income and minority neighborhoods [33-37]. This finding, if replicated in other studies, may explain a part of the socioeconomic disparities in eating behaviors that have been observed. Yet it also raises a question: why might it be more attractive to advertise obesigenic foods in these vulnerable neighborhoods?

This conjecture may offer important insights into the causes that underlie socioeconomic disparities in dietary behaviors. If cognitive load potentiates the effects of obesigenic food advertising, then disparities in stress and cognitive load could translate into disparities in healthy eating behaviors.

This study tests whether food advertising has a significant effect on the types and quantity of food chosen in a free-choice environment, and explores how these effects of advertising differ when cognitive load is experimentally manipulated.

A secondary analysis presents these results stratified by the socioeconomic status of the participants.

## Methods

This study used a 2x2 factorial design, with both advertising and cognitive load experimentally manipulated, to test the effects of food advertising on food choice overall and among subsets of participants who received either high-cognitive-load or low-cognitive-load tasks.

### Sample

Participants were students at UCLA, recruited through posters, ads in the campus newsletter, and a campus-wide student participant pool maintained by the Anderson Behavioral Lab, a part of the UCLA Anderson School of Management. All willing students aged 18 or older and without any major self-reported health problems (such as asthma, diabetes, heart disease or depression) were eligible to participate. Participants were told that they would be participating in a study of “television viewing and short-term memory”. Those who completed the study were given a $10 gift certificate to on-campus stores and restaurants.

Participants who met the above inclusion criteria were randomly assigned to one of four groups:

1. High cognitive load + food advertising

2. Low cognitive load + food advertising

3. High cognitive load + non-food advertising

4. Low cognitive load + non-food advertising

### Procedures

Participants were invited in groups of 20 to view prerecorded movie segments interspersed with advertising. Each session included, in this order, a brief introduction to the study, 45 minutes of viewing, a brief break for snacks (including water and soda options), and the completion of a survey of demographic and other information. The entire session typically lasted just under an hour. Eligible enrollees were asked to enroll for a particular study session via an online scheduling system. The study slots were then randomly assigned to one of the four experimental arms, ensuring only that approximately equal number of sessions were conducted in morning, noon and afternoons.

The viewing consisted of a 3 blocks of content and each block included three 30-second commercials. Each block began with an introductory or transition screen displayed for 15 seconds, an introductory announcement such as that seen in movie theaters requesting that people silence their cell phones, and one, 30-second commercial. This introductory material— 45 seconds total—was followed by a 6-to-7-minute segment excerpted from a movie or television show, a second 30-second commercial break, a second movie or TV segment, and finally one more 30-second commercials. For those participants assigned to the food-advertising arm, the 2 of the 3 commercials were for an obesigenic food product—potato chips, chocolate candy and sugary soda. The order in which food commercial was introduced within each block varied. Each participant assigned to the food-advertising arm accordingly was exposed to 6, 30-second food commercials. In the intervention arm, 1 of the 3 commercials was for irrelevant products (such as cars, sneakers or cell phones). Those assigned to the control-advertising arm saw the same irrelevant commercials as in the food-advertising arm, and in addition saw additional non-food commercial in the place of food commercial in each block. Participants in both arms saw the same number of commercials and the same TV and movie programming. The movie and TV excerpts were chosen to be entertaining, but not highly stimulating, and to avoid mention of food, eating, or obesity-related topics. The same TV and movie excerpts were used in both arms. Additional file 1 reports the full schedule of viewing in both arms.

There were two parts to the cognitive task, a task involving remembering a number and a task involving tracking information on screen.

Immediately before the introductory screen of the third block, participants were shown a number for 7 seconds and asked to memorize the number. Participants were asked not to write the number down and were told that they would be asked to record the number on their final survey. Those assigned to the high-cognitive-load condition were asked to remember a 7-digit number. Those assigned to the low-cognitive-load condition were asked to remember a 2-digit number. These cognitive tasks were chosen because of their similarity to a previous experiment involving cognitive load and food choice [30]. The specific numbers are reported in Additional file 1. The information task demanding high cognitive load required the participants to mentally keep track of the number of times a particular word was uttered in a movie segment. (For example, in the sector showing ‘Duck Dynasty’, the participants were asked to count the number of time the word duck is uttered by any of the actors.) At the end of that segment, they were required to write down the total count on the task answer sheet given to them.

In addition to memorizing a 2-digit number, the low cognitive load task was to answer a simple question per segment. (For example, in the ‘Duck Dynasty’ segment, the question asked about the show’s location, which was mentioned multiple times during the segment.)

At the beginning of the study, participants were informed of the study purpose and protocol and provided their verbal consent to participate. The study protocol was approved by the UCLA Institutional Review Board, approval #12-000323.

### Variables

A variety of snack and drink items were made freely available to the participants during a break that took place after the viewing and before the survey. Participants were told that there were snacks on the table on one side of the room, and that they were invited to help themselves. These items included water, small bags of sliced apples, small bags of trail mix, granola bars, Coca Cola, small bags of M&M’s, Reese’s Peanut Butter Cups, Hershey’s Kisses, and Lay’s Potato Chips. Ads for Coca Cola, Hershey’s Kisses, M&M’s and Lay’s Potato Chips were included as part of the experimental manipulation in the food-advertising arm. Because no ads were presented for water, apples, trail mix or granola, these items were deemed healthy, with the candy, soda, and potato chips deemed unhealthy. These labels are intended as convenient descriptors only, as it is true that excessive consumption of, say, trail mix, would not be healthy.

Two main outcome variables were assessed: the number of snack items chosen and the total count of calories of food that was chosen. These outcomes were chosen to reflect each of the two distinct dimensions of food-related choices: the type of food chosen and the quantity chosen. Actual consumption of food was not a behavioral target of the experiment and was not observed in the study. Within each of these outcomes, the analysis separately tracks the number of calories from healthy and unhealthy items and the number of healthy and unhealthy items chosen.

The number and types of snack items (including drinks) were observed and recorded by one of the coauthors (SS). Discrete video recording of the snacks area permitted accurate assessment of the items taken by each study participant. Calorie counts were available for each of the healthy and unhealthy items.

The final questionnaire included questions on age, gender, year in school, major, exercise and sleeping habits, fast food consumption, soda consumption, and. television viewing habits. Following previous work on economic disparities in obesity, students were asked to provide the zip code of their parents’ address as a proxy for socioeconomic status [38]. Data from the 2011 American Community Survey, collected by the US Census, were used to determine the average income for each zip code. Participants were dichotomized into high vs low socioeconomic status according to whether the average income in their home zip code is above or below the within-sample median. Foreign students (N = 48) were dropped from these analyses.

### Statistical analysis

The number of unhealthy snack items chosen is count data, with a Poisson distribution. A likelihood ratio test failed to reject the assumption of equidispersion (i.e., that the conditional mean and conditional variance of the outcome are equal; p-value = 0.38), suggesting that poisson is preferred to a negative-binomial regression. The Vuong test revealed no evidence of zero-inflation. Accordingly, the assumptions of Poisson regression could not be rejected and hence, it was the preferred model.

The Poisson regression was first conducted in the whole sample to test the main effects of advertising. To gain some purchase on the statistical meaning of the differences in the effects of advertising between high-cognitive-load and low-cognitive-load conditions two tests were conducted. First, an advertising-cognitive-load interaction term was added to the regressions and its significance was tested. If the coefficient on this term were significant, it would indicate that the analysis could reject the null hypothesis of no effect-modification of advertising by cognitive load.

Second, an equivalence test was conducted [39], using a two one-sided test (TOST) with a delta of 50 kcal for the total calories and 25% for the number of unhealthy snacks. The purpose of an equivalence test is to determine whether the observed point estimate, along with its entire confidence interval, is contained within a specified margin around some anchor, often either zero or some other known quantity. Unlike a statistical significance test, the purpose of an equivalence test is to test the magnitude of difference between an estimate and some other quantity. In this analysis, the question is whether the effects of advertising can be said to be of similar magnitude in a high-cognitive-load and a low-cognitive-load condition. Note that significant differences and equivalences are conceptually distinct: estimates in these two conditions could be statistically significantly different and yet equivalent; not statistically significantly different and yet not equivalent; or any other combination. The equivalence used a one-sided test of whether the interaction of cognitive load and advertising was associated with a change in either total calories or the number of unhealthy snacks of less than 50 calories or less than 25%, respectively.

The sample was then split into sub-samples of high-cognitive-load and low-cognitive-load, and the Poisson regression was conducted in each sample separately to test the effects of advertising under these distinct conditions.

Finally, as a secondary analysis, the samples were further stratified within cognitive-load arms by socioeconomic status, divided at the sample median (excluding the foreign-born participants). Again, the Poisson regression was conducted, this time in 4 distinct sub-samples of the data.

In each regression, the participant’s status in the food-advertising or non-food advertising arm is the only regressor.

The number of calories is a normally distributed variable, but truncated on the left at zero. With this distribution for the dependent variable, Tobit regression is indicated. As for the first outcome, the number of calories chosen was analyzed first in a Tobit regression of the whole sample, with tests for effect modification and equivalence (with a delta of 50 kcal), and then in a stratified regression by cognitive load and finally in a sub-analysis in which the sample was further stratified by socioeconomic status.

All analyses were carried out using Stata 10.1.

### Sensitivity analyses

A small number (N = 3; <1%) of the participants were observed either to have written their number down when they were asked to remember it, or recalled a number that was substantially different than the number they had been given. The results reported here were analyzed without correcting for this protocol violation. However, an analysis in which these participants were dropped (not reported here) produced highly similar results.

Several additional analyses were conducted to test the robustness of the results to alternative specifications. These analyses included ordinary least squares regression instead of Poisson or Tobit, and analyses that were adjusted for the gender, parental SES, year in college, foreign citizenship and past food habits of the participant. All analyses produced results that were highly similar to those reported here.

## Results

Table 1 presents the descriptive statistics of the sample. Consistent with the randomization of the participants, there are few meaningful differences across the groups.

**Table 1:** Descriptive statistics

	**Food advertisement**		**Non-food advertisement**		**p-value of test of equality across groups**	
	**High cognitive load**	**Low cognitive load**	**High cognitive load**	**Low cognitive load**	**Food vs Non-food adverts**	**High vs Low cog. load**
N	92	89	86	84		
Female (%)	67%	72%	79%	80%	0.04	0.57
Foreign (%)	12%	11%	16%	15%	0.24	0.84
High Income (%)	41%	36%	47%	43%	0.25	0.39
Low Income (%)	45%	48%	36%	39%	0.10	0.51
Income missing (%)	14%	16%	17%	18%	0.49	0.79
Quality of diet (1-5)^a^	2.75	2.63	2.85	2.74	0.25	0.20
(Standard deviation)	*(0.86)*	*(0.80)*	*(0.77)*	*(0.91)*		
Weekly Fast Food consumption (times/week)	1.24	1.80	1.26	1.67	0.80	0.02
(Standard deviation)	*(1.47)*	*(2.35)*	*(1.32)*	*(2.20)*		
Regular Excercise (%)	43%	56%	52%	56%	0.25	0.06
Year of expected Graduation	2014.6	2014.5	2014.3	2014.6	0.46	0.46
(Standard deviation)	*(1.17)*	*(1.32)*	*(1.25)*	*(1.21)*		

^a^Likert scale: Excellent (5); Very Good (4); Good (3); Fair (2); Poor (1).

**Figure 1:**
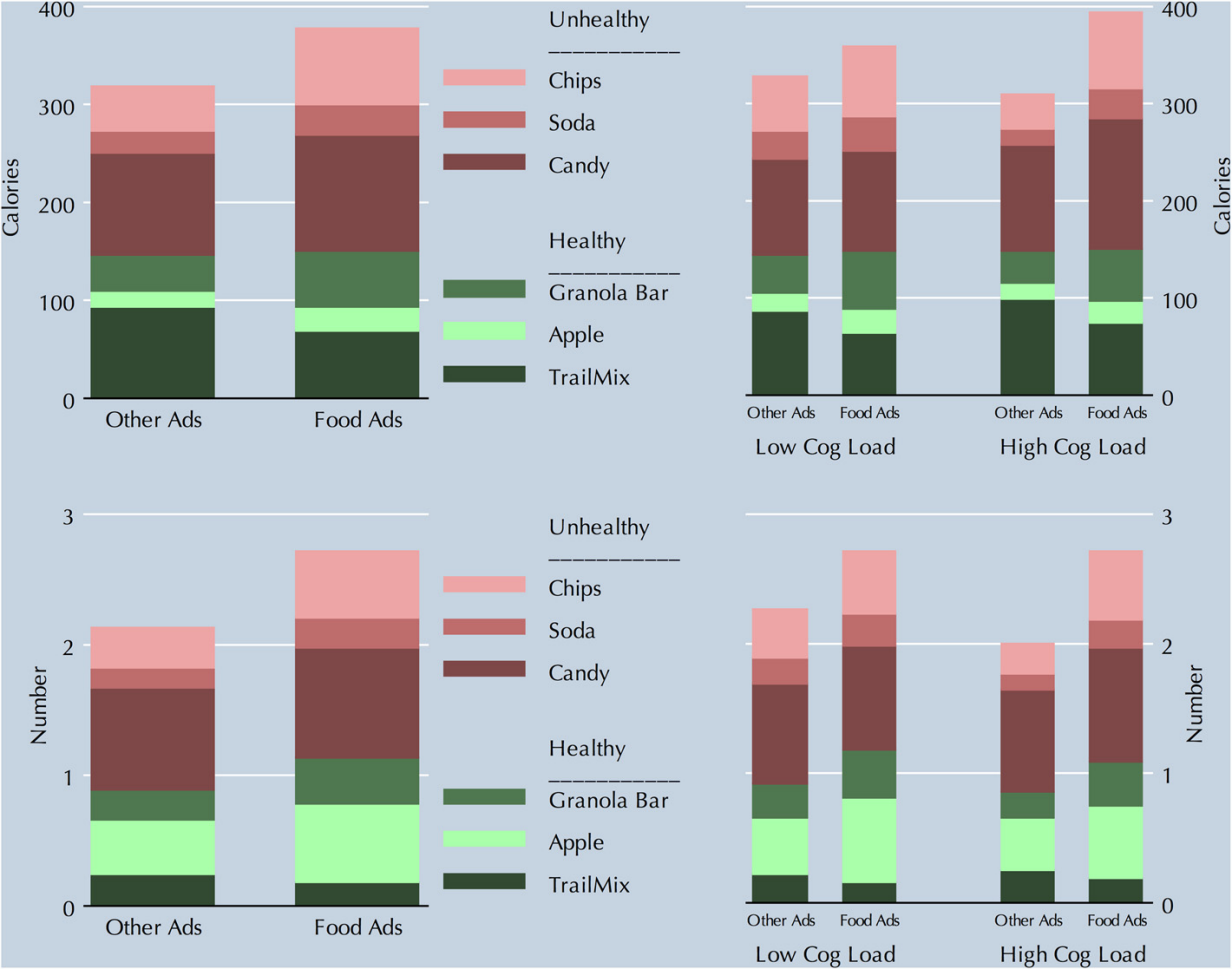
Calories and Number of snacks by experimental arm.

Figure 1 presents the unadjusted results graphically. The top panel reports results in terms of calories, and the bottom panel in terms of the number of snacks chosen. Results are broken down by individual food type, within the categories of healthy and unhealthy food. In the left pane is the simple comparison of food choices in the non-food-advertising arm and the food-advertising arm; in the right pane the effect modification by cognitive load is presented. In all comparisons, more food was taken in the food-advertising arm than in the non-food advertising arm. For calories, most of the increase was among the unhealthy foods, with the largest percentage increases for soda and chips. For number of items, there were large increases in the unhealthy foods, again with proportionately large increases for soda and chips. However, food advertising was also associated with an increase in the selection of apples, and with a decrease in selection of trailmix.

Table 2 presents a formal statistical analysis of these results. Three models are presented: the main effect of advertising, the effects of advertising controlling for the set of covariates included in Table 1, and the effects of the advertising-cognitive-load interaction. Each model is executed for the total number of calories and the number of unhealthy snacks.

**Table 2:** Effect of food advertising on the type and quantity of food chosen

	**Number of calories**^**a**^		**Number of unhealthy snacks**^**b**^	
	**Coefficient**	**[95% Confidence interval]**^**c**^	**Coefficient**	**[95% Confidence interval]**^**c**^
*Model 1: Food advertising alone*				
Food advertising	**65**	**[10 – 121]**	**1.28**	**[1.07 – 1.53]**
*Model 2: Food advertising with additional controls*^d^				
Food advertising	**67**	**[11 – 122]**	**1.28**	**[1.07 – 1.53]**
Female	-2	[–66 – 62]	1.00	[0.82 – 1.23]
Foreign	**211**	**[53 – 369]**	**1.99**	**[1.16 – 3.43]**
High income	130	[–18 – 278]	1.44	[0.85 – 2.45]
Low income	**178**	**[28 – 327]**	**1.80**	**[1.05 – 3.07]**
Diet quality	19	[–17 – 54]	1.03	[0.92 – 1.15]
Fast food	-3	[–18 – 12]	1.00	[0.95 – 1.04]
Regular exercise	-39	[–97 – 19]	0.91	[0.76 – 1.10]
Year degree expected	1.6	[–21 – 24]	1.03	[0.95 – 1.10]
*Model 3: Food advertising with interaction effect*				
Food advertising	36	[–43 – 114]	1.14	[0.89 – 1.47]
High cognitive load	-22	[–101 – 57]	0.85	[0.65 – 1.11]
Food advertising + High cognitive load	59	[–51 – 169]	1.25	[0.88 – 1.79]
**Equivalence tests:**				
Interaction ≤ 50 kcal		p-value = 0.56		
Interaction effect has a rate ratio ≤ 1.25				p-value = 0.50

^a^Tobit regression.

^b^Poisson regression.

^c^Bolded coefficients are significant at the 5% level.

^d^See Table 1 for variable definitions.

Those exposed to food advertising took a set of snacks with 65 more calories than those exposed to non-food advertising, and this difference is significant (p-value = 0.02; 95% CI: 10-121). Again, neither the effect modification by cognitive load nor the equivalence test achieved significance (p-values of 0.30 and 0.56, respectively).

Results of the Poisson estimation of the number of unhealthy snacks are reported with exponentiated coefficients, which can be interpreted as a percentage increase relative to the reference category. The exponentiated coefficient (rate ratio: RR) in the pooled regression is 1.28 (95% CI: 1.07 – 1.53). That is, those in the food-advertising group chose 28% more unhealthy snacks than those in the non-food advertising group. Neither the effect-modification of advertising by cognitive load nor the equivalence test was significant (p-values of 0.22 and 0.50, respectively). Low-income and foreign students chose more snacks and more total calories than non-foreign and high-income students. No other covariates were significant, and—as expected in a randomized experiment—the covariates collectively do not moderate the main effects.

The results of the Tobit regressions of number of calories are reported in Table 3. In the low-cognitive-load group the effect was not significant for all calories, calories from healthy foods and calories from unhealthy foods. In the high-cognitive-load group the effect was significant for total calories and calories from unhealthy foods. Those in the food-advertising group chose a set of snacks with 94 more calories than the non-food advertising group (95% CI: 19-169); and their choice of unhealthy foods had 107 more calories than those of the non-food-advertising group (95% CI: 33-181). Accordingly, all of the additional calories associated with food advertising were from unhealthy foods.

**Table 3:** Effect of food advertising on the number of calories chosen

		**All calories**		**Healthy calories**		**Unhealthy calories**	
	**N**	Coeff*	***p-value***	Coeff*	***p-value***	Coeff*	***p-value***
		**[95% CI]**		**[95% CI]**		**[95% CI]**	
*Full sample*	**351**	**65 [10 – 121]**	***0.02***	**15 [–34 – 65]**	***0.55***	**70 [19 – 122]**	***0.01***
*Stratification by experimentally manipulated cognitive load*							
Low-cognitive-load sub-sample	173	36 [–46 – 118]	*0.39*	15 [–57 – 88]	*0.68*	34 [–38 – 105]	*0.36*
**High-cognitive-load sub-sample**	**178**	**94 [19 – 169]**	***0.01***	15 [–53 – 83]	*0.67*	**107 [33 – 181]**	***0.01***
*Sub-stratifications by parental SES*							
Low cognitive load							
High SES	68	69 [–75 – 213]	*0.34*	58 [–70 – 185]	*0.37*	38 [–78 – 154]	*0.51*
Low SES	76	-45 [–153 – 63]	*0.41*	-52 [–147 – 43]	*0.28*	16 [–99 – 132]	*0.78*
High cognitive load							
High SES	78	34 [–92 – 160]	*0.59*	28 [–79 – 134]	*0.61*	27 [–105 – 160]	*0.68*
Low SES	72	90 [–22 – 203]	*0.11*	-51 [–163 – 60]	*0.36*	**143 [37 – 249]**	***0.01***

*The coefficient (Coeff) is the difference in total calories chosen by those in the food advertising group from the total chosen by those in the non-food-advertising group. [95% Confidence Intervals (CI) in brackets].

**Note**: For each outcome, the results of 7 separate Tobit regressions are reported. In each regression the intervention status is the only variable. Significant results in bold.

The secondary stratified analyses using socioeconomic status revealed no statistically significant results, except that below-median-SES participants in the high-cognitive-load plus food-advertising arm chose snacks with 143 more calories than those in the high-cognitive-load plus non-food-advertising arm (95% CI: 37-249).

Stratified results of the Poisson regressions of the total number of snacks, unhealthy snacks, and healthy snacks are presented in Table 4. In the low-cognitive-load group, the effect of food advertising is not significant for all snacks, healthy snacks and unhealthy snacks. In the high-cognitive-load group, those exposed to food advertising chose 28% more total snacks and 43% more unhealthy snacks (rate ratio 95% CIs: 1.07-1.54 and 1.11 – 1.85, respectively). The effect on healthy snacks was not significant.

**Table 4:** Effect of food advertising on the number of snacks chosen

		**All snacks**		**Healthy snacks**		**Unhealthy snacks**	
	**N**	Rate ratio* [95% CI]	***p-value***	Rate ratio* [95% CI]	***p-value***	Rate ratio* [95% CI]	***p-value***
*Full sample*	**351**	**1.23 [1.09 – 1.40]**	***0.01***	1.19 [1.00 – 1.42]	*0.06*	**1.28 [1.07 – 1.53]**	***0.01***
*Stratification by experimentally manipulated cognitive load*							
Low-cognitive-load Sub-sample	173	1.18 [0.99 – 1.41]	*0.06*	1.22 [0.96 – 1.57]	*0.11*	1.14 [0.89 – 1.47]	*0.29*
**High-cognitive-load Sub-sample**	**178**	**1.28 [1.07 – 1.54]**	***0.01***	1.15 [0.89 – 1.48]	*0.28*	**1.43 [1.11 – 1.85]**	***0.01***
*Sub-stratifications by parental SES*							
Low cognitive load							
High SES	68	**1.46 [1.10 – 1.95]**	***0.01***	**1.81 [1.20 – 2.71]**	***<0.01***	1.18 [0.78 – 1.77]	*0.44*
Low SES	76	0.95 [0.73 – 1.23]	*0.70*	0.88 [0.61 – 1.27]	*0.50*	1.02 [0.71 – 1.47]	*0.90*
High cognitive load							
High SES	78	1.15 [0.87 – 1.52]	*0.32*	1.29 [0.88 – 1.88]	*0.19*	1.01 [0.67 – 1.52]	*0.59*
Low SES	72	1.26 [0.95 – 1.68]	*0.11*	0.84 [0.56 – 1.27]	*0.41*	**1.84 [1.22 – 2.78]**	***<0.01***

*Ratio of unhealthy snacks chosen by those in the food-advertising group to those in the non-food-advertising group.

**Note:** For each outcome, the results of 7 separate Poisson regressions are reported. In each regression the intervention status is the only variable. Coefficients can be interpreted as the percentage increase in number of unhealthy snacks chosen in the food advertising group over the number chosen in the non-food advertising group (i.e., 1.28 implies that the food-advertising group chose an average of 28% more unhealthy snacks than non-food advertising group). Significant results are in bold.

The secondary analyses further stratifying these results by parent socioeconomic status revealed a significant effect among those in the high-cognitive-load group with below-sample-median income. In this group, the effect of food advertising was an 84% increase in the number of unhealthy snacks chosen (rate ratio 95% CI: 1.22 – 2.78), and this effect was significantly different than among the above-sample-median group. Those in the high-cognitive-load group with above-median SES had increases of 46% and 81%, respectively, in the number of snacks overall and the number of healthy snacks chosen (95% CIs” 1.10-1.95 and 1.20-2.71, respectively). The effect of food advertising was not significant in all other groups, and there were no other significant effect modifications by SES in any of the other regressions.

## Discussion

There is a clear qualitative difference between the high-cognitive-load group, for whom advertising has a large and statistically significant effect, and the low-cognitive-load group, for whom advertising has a smaller, and statistically insignificant effect. These differences appear to be magnified by the participant’s socioeconomic status, with low-SES individuals more susceptible to the effects of advertising than high-SES individuals.

These study results are similar to those found in Harris, Bargh, and Brownell (2009), which included 4 food advertisements (20 seconds each, as opposed to 3, 30-second advertisements here). Although the coding of the outcome in the two studies was too different to permit a direct comparison, the Harris et al. study found that those in the food-advertising group consumed 0.44 standard deviations more than in the control group, an effect of a broadly similar magnitude to that estimated here.

We are unaware of any study in the literature that examines whether the effect of advertising can be enhanced by cognitive load. Two studies have noted an interaction between restrained eating and either cognitive load [31] or advertising [9] on increased calorie choice in experimental settings. Another study found that emotional setbacks like a favorite sports team losing an important match can trigger overeating [40].

Our results suggest that the conjoint presence of both heavy cognitive load and food advertising might lead to significantly worse food choices. Other research has shown people often watch television while distracted in some way, for example by multi-tasking. To the extent that such multi-tasking induces cognitive load, the research here suggests that it may exacerbate the effects of advertising. In addition, evidence suggests that television viewing in childhood and adolescence has sustained effects into adulthood [13,41]. If low-SES children are more likely to be exposed to television advertising for obesigenic foods, the longevity of the effect may explain some of the results here. Participants’ prior exposure to food marketing was not assessed here, and this is a limitation of the present research.

Food advertising is much discussed in the public health literature, but most of the popular discussion around food advertising seems to focus on children [14,42-44], while scant attention is paid to adults. This study contributes to a very small but important body of literature that suggests that the effects of advertising are not limited to children.

The results of this study reinforce the research consensus that advertising is a potent force in food choice. Americans tend to resist calls for restrictions on marketing by invoking values around freedom. Yet it is worth closely examining the meaning of free choice [45]. In this experiment all participants were equally free to choose, and yet the study authors were able to manipulate this freedom, influencing choices through experimental conditions. In the world outside the lab, choices can also be manipulated [46]. Carefully studied experience from a ban on advertising to children in Québec shows that such a ban is effective in promoting healthier eating [47].

Previous research has found that those of low socioeconomic status may be especially likely to suffer from stress [48-50]. For example, one recent study in which race/ethnicity was strongly correlated with education and income, found that African-Americans had experienced an average of 1.92 stressors and American-born Latinos 1.90, against only 1.12 events for Whites [51]. It may be that the daily hassles and stressors experienced by minority and low-income communities operate in a similar way to the experimentally induced cognitive load described here. If so, that would suggest that people so exposed might be more than usually susceptible to the effects of food advertising.

Eating behavior is strongly influenced by cultural and environmental factors [52]. The results presented here raise the possibility that food marketing may be more potent in low-income neighborhoods than in high-income ones. Future research should attempt to replicate and extend this research to further examine patterns related to cognitive burden and socioeconomic factors.

### Limitations

Participants in this study were all students at a top-ranked university, which may limit the external generalizability of the study. UCLA is one of the most ethnically and economically diverse universities in the country [53] and has the highest proportion of students receiving Pell Grants of any major university, an important indicator of economic diversity [54]. All the same, many of the results on food choice obtained to date have been conducted among college students, and research in the community would enhance confidence in the generalizability of the results.

Socioeconomic status in this study was measured by a proxy of parental zip code, which is clearly an imperfect measure. In the US, a zip code includes approximately 7,000-10,000 people. Because housing costs in the US tend to follow geographic patterns, zip codes tend to have some degree of economic homogeneity. Yet this homogeneity is not absolute, and there can be variations of income within zip code. In this data set the standard deviation of parental SES as measured by zip code proxy was 38% of the mean. This measure was used because the socioeconomic status of college students is hard to operationalize. An advantage of replicating these results in the community would be the ability to capture more reliable measures of socioeconomic status.

This research is motivated by the possibility that chronic cognitive load enhances the effect of chronic exposure to food advertising. Yet in the confines of this experiment neither chronic cognitive load nor chronic exposure to food advertising could be experimentally manipulated. It could be that the effects of chronic exposures are either greater or lesser than the very brief and relatively small doses manipulated in this experiment. Given how pervasive and profound both cognitive load and food advertising are in American society, other methods besides experimental manipulation will be necessary to tease out the causal roles and interactions of these two factors on eating behaviors.

## Conclusion

“Marketing works”. These opening words of the Institute of Medicine’s report on food marketing to children [14] apply to adults as well as to children. These study results raise the possibility that food marketing may have disparate effects across different populations, disproportionately influencing the eating behaviors of some of the most vulnerable subgroups and potentially contributing to disparities in diet and in related health outcomes.

## Abbreviations

SES: Socio-economic status; RR: Rate ratio; CI: Confidence interval.

## Competing interests

Both authors declare that they have no competing interests.

## Authors’ contributions

FZ conceived the idea for the research. SS and FZ together designed the specific aspects of the study protocol. SS recruited the participants and conducted the experiments. SS prepared and cleaned the data, and FZ conducted the analyses. SS reviewed the analyses. Both FZ and SS drafted the final document. Both authors read and approved the final manuscript.

## Pre-publication history

The pre-publication history for this paper can be accessed here:

http://www.biomedcentral.com/1471-2458/14/342/prepub

## Supplementary Material

Additional file 1 Movie and Advertisement Sequence. Click here for file
