# Supplementary material for: The effects of food advertising and cognitive load on food choices
Source: BMC Public Health. 2014 Apr 10;14:342. doi: 10.1186/1471-2458-14-342 (PMC4021209; doi:10.1186/1471-2458-14-342)
Supplement: Additional file 1 — Movie and Advertisement Sequence. [file 1471-2458-14-342-S1.docx]

# Additional file 1 – Movie and Advertisement Sequence

|  | **Screen Time (Seconds)** | **High/ food** | **low/ food** | **high/ other** | **low/ other** |
| --- | --- | --- | --- | --- | --- |
| **Block 1** | | | | | |
| Introduction Screen | 15 | “Introductory Screen” | | | |
| Pre movie announcements | 30 | “Please silence your cell phone” animation | | | |
| First Ad | 30 | M & M’s (“Sexy & You Know it”) | | Hyundai Equus (driving through city) | |
| Movie 1 - comedy | 360 | Duck Dynasty  (title sequence & character introductions) | | | |
| Second Ad | 30 | Coca Cola Polar Bears | | Head and Shoulders (Michael Phelps) | |
| Movie 1 - comedy | 360 | Duck Dynasty  (continuation: more character introductions) | | | |
| Third Ad | 30 | Sketchers (Dog Racing) | | | |
| **Block 2** | | | | | |
| Introduction Screen | 15 | “Revenge” | | | |
| Pre movie announcements | 30 | “Please silence your cell phone” old couple on bench | | | |
| First Ad | 30 | Lay’s Potato Chips (“One taste and you’re in love”) | | Scotts Turf Builder (lawn care ad) | |
| Movie 2 - drama | 420 | Revenge: From the Beginning  (Title Sequence and character intro) | | | |
| Second Ad | 30 | Jeep Grand Cherokee (driving through city and along coast) | | Kia Optima (Blake Griffin) | |
| Movie 2 - drama | 360 | Revenge: From the Beginning  (continuation: polo match, etc) | | | |
| Third Ad | 30 | Pepsi (“Whatever Lola Wants Lola Gets”) | | Jeep Grand Cherokee (driving through city and along coast) | |
| Cognitive Task Intro | 15 | “8371572” | “58” | “8371572” | “58” |
| **Block 3** | | | | | |
| Introduction Screen | 15 | “The Apartment” | | | |
| Pre movie announcements | 30 | “Please silence your cell phone” animation | | | |
| First Ad | 30 | Old Navy (Mr. T) | | | |
| Movie 2 – classic comedy | 375 | The Apartment (1960)  (opening scenes) | | | |
| Second Ad | 30 | Doritos (Pug Attack) | | Jeep Wrangler (driving down a snowy mountain) | |
| Movie 2 – classic comedy | 410 | The Apartment (1960)  (continuation: Baxter is asked to lay in more liquor, etc) | | | |
| Third Ad | 30 | Hershey’s chocolate (animated chocolate figures dancing) | | JBL speakers  (Paul McCartney in rainy city) | |
| Concluding Screen | 15 | “The End” | | | |
